# Unusual presentation of giant cell tumor originating from a facet joint of the thoracic spine in a child: a case report and review of the literature

**DOI:** 10.1186/1752-1947-7-178

**Published:** 2013-07-05

**Authors:** Koopong Siribumrungwong, Boonsin Tangtrakulwanich, Anupong Nitiruangjaras

**Affiliations:** 1 Department of Orthopedic Surgery, Faculty of Medicine, Prince of Songkla University, Hat Yai, Songkla 90110, Thailand; 2 Department of Orthopedic Surgery, Faculty of Medicine, Prince of Songkla University, Hat Yai, Songkla 90110, Thailand; 3 Department of Pathology, Faculty of Medicine, Prince of Songkla University, Hat Yai, Songkla 90110, Thailand

**Keywords:** Giant cell tumor, Spine, Synovium

## Abstract

**Introduction:**

Giant cell tumor of the synovium is a common benign lesion that frequently occurs at the tendon sheaths in the hand; it is usually found in adults over 30 years old. It is related to pigmented villonodular synovitis. Giant cell tumor of the synovium or pigmented villonodular synovitis has been described rarely in the axial skeleton especially in the thoracic vertebrae of a child.

**Case presentation:**

A previously healthy 7-year-old Thai girl presented with back pain and progressive paraparesis and was unable to walk for 1 month. She had weakness and hyperreflexia of both lower extremities. Magnetic resonance imaging showed a well-defined homogeneously and intensely enhanced extradural mass with cord compression at T4 to T7 levels. The patient underwent laminectomy at T4 through to T7 and total tumor removal. Permanent histopathologic sections and immunostains revealed a giant cell tumor of the synovium. Postoperative neurological status recovered to grade V. Magnetic resonance imaging at the 1-year follow-up showed no recurrence and there was no clinical recurrence at the 2-year follow-up.

**Conclusion:**

We report an extremely rare case of giant cell tumor in the epidural space that extended from a thoracic facet joint. The tumor was removed successfully through laminectomies. Although giant cell tumor of a facet joint of the thoracic spine is very rare, it must be considered in the differential diagnosis for masses occurring in the epidural space in a child. Total tumor removal is the best treatment. Careful monitoring of recurrence can achieve a good clinical outcome.

## Introduction

Giant cell tumor of the tendon sheath usually originates from the synovial membrane of tendon sheaths, bursa, and joints [1]. Pigmented villonodular synovitis (PVNS), nodular tenosynovitis and giant cell tumor of the tendon sheath are related lesions with common histologic patterns [2] but different anatomic origins and clinical presentations [3], so unification of these entities has been suggested [4]. According to the World Health Organization, tenosynovial or synovial giant cell tumors are subtyped into localized and diffused types [1]. The localized type (giant cell tumor of the synovium) is encapsulated, extra-articular and commonly found in the tendon sheaths of the fingers whereas the diffuse type is non-encapsulated, intra-articular and commonly found in the knee joint, and is designated as “PVNS”. The main difference between PVNS and giant cell tumor of the tendon sheath is the intra-articular growth in the former group and the extra-articular growth in the latter group.

Synovial-type giant cell tumors or PVNS rarely arise in the region of the axial skeletal system. Most reports of synovial-type giant cell tumors in the axial skeletal system published to date have been designated “PVNS” and are usually involved in the adult lumbar or cervical spine rather than the thoracic spine [5-7]. To date less than 50 examples of synovial-type giant cell tumors or PVNS of the spinal region are documented in the English literature [8]. Only 11 cases involved the thoracic spine and only two cases were related to children [6-13] (Table 1).

**Table 1:** Summary of published data on giant cell tumor of the synovium of the thoracic vertebrae

**Authors and year of publication**	**Number of cases**	**Age (years)**	**Gender**	**Level**	**Facet involvement**	**Epidural involvement**	**Treatment**
Kuwabara *et al.* 1992 [10]	1	25	F	T8–T11	+	+	STR and radiation
Clark *et al*. 1993 [9]	1	23	M	T7–T8	+	+	GTR
Giannini *et al*. 1996 [6]	1	40	M	T11	NA	NA	GTR
Furlong *et al.* 2003 [7]	1	21	F	T4–T5	+	+	GTR
Doita *et al.* 2005 [13]	1	26	M	T8–T11	+	+	GTR
Motamedi *et al.* 2005 [12]	4	21	F	T4–T5	+	+	NA
		7	F	T2–T3	+	+	NA
		36	F	T5–T6	+	+	NA
		30	M	T5–T6	–	+	NA
Hansen *et al.* 2007 [11]	1	17	M	T6–T7	+	+	GTR
Gupta *et al.* 2008 [8]	1	9	F	T8–T9	+	+	GTR

GTR, gross total resection; NA, not available; STR, subtotal resection; M, male; F, female.

We describe a case of a 7-year-old Thai girl with giant cell tumor of the synovium with an extremely rare presentation in the thoracic spine.

## Case presentation

**Figure 1:**
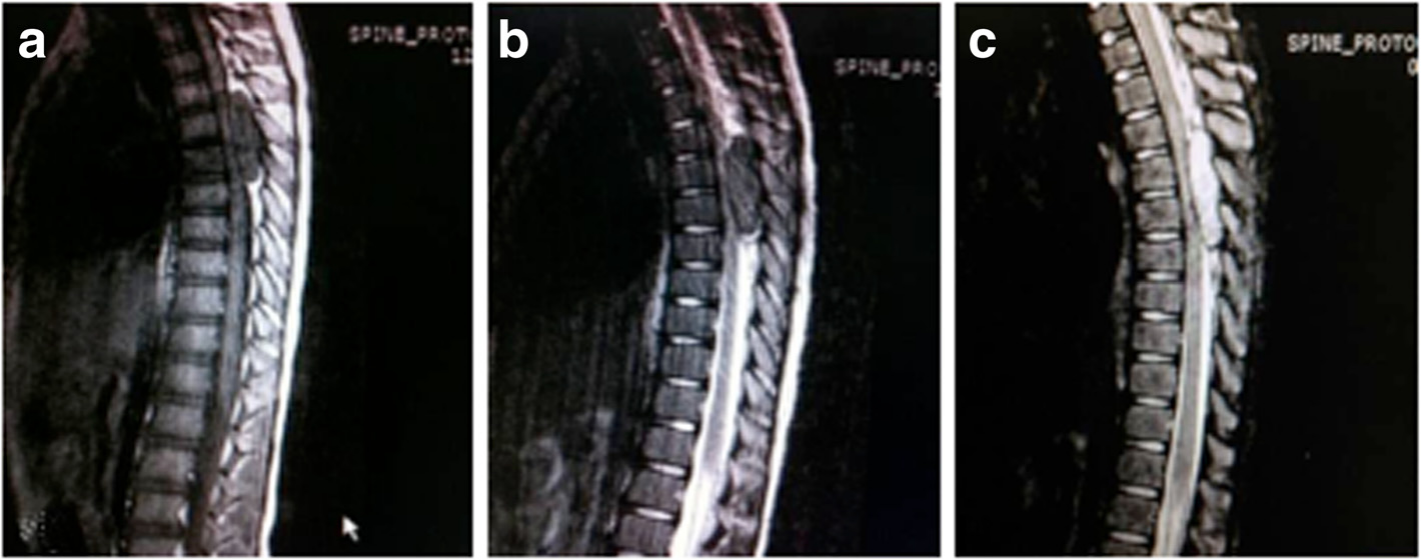
**Preoperative sagittal magnetic resonance imaging. a**. Preoperative sagittal T1-weighted magnetic resonance imaging of the dorsal spine showing extradural mass in posterior aspect of dorsal canal approximately 1.0 × 1.4 × 4.0cm in size along T4 to T7 levels with hypointense signal. **b**. Preoperative sagittal T2-weighted (T2W) magnetic resonance imaging showing extradural mass with intermediate intense signal. **c**. Preoperative sagittal post-contrast T2W showed intense homogeneity with significant enhancement.

**Figure 2:**
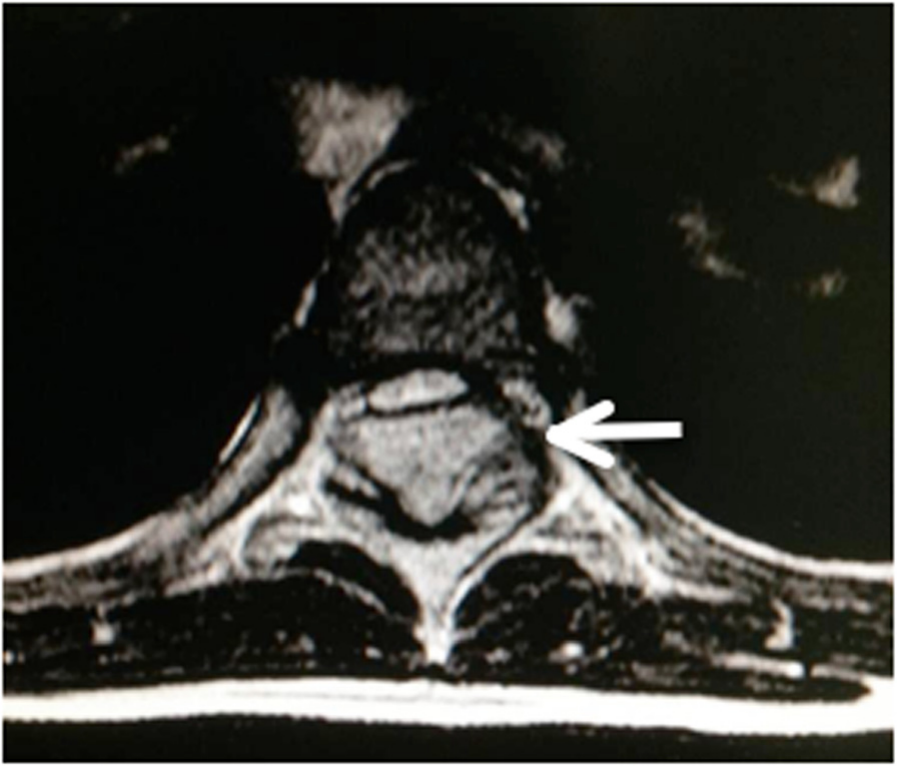
**Preoperative axial magnetic resonance imaging.** Axial T2-weighted image demonstrating tumor extension from the left facet joint of T5 to T6 (arrow).

**Figure 3:**
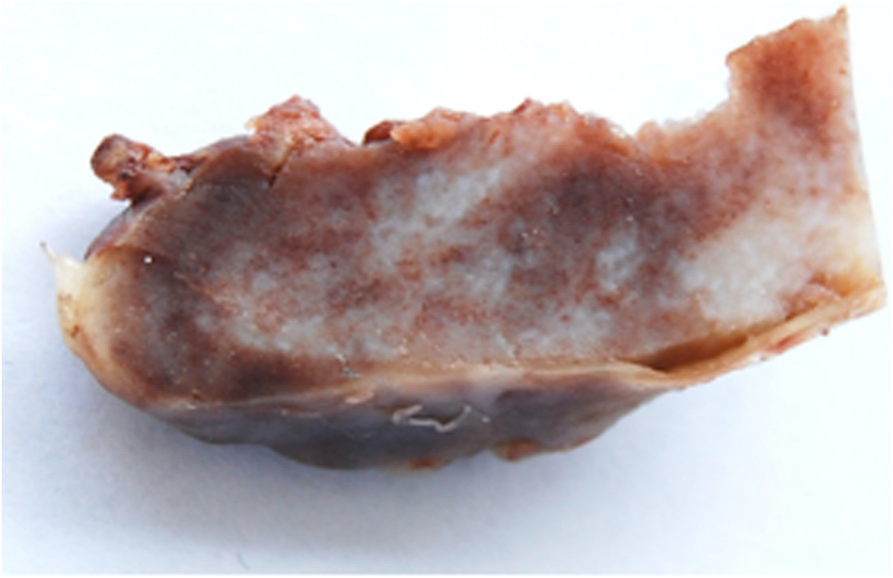
**Gross pathology of tumor.** It was a well-capsulated firm to hard mass measuring 1.0 × 1.5 × 4.0cm in diameter. Cut surfaces showed white-yellow tissue and a tiny bone component at the capsule.

A previously healthy 7-year-old Thai girl presented with back pain, progressive paraparesis and was unable to walk for 1 month. The physical examination showed no scoliosis, but did show weakness of her lower extremities grade III and hyperreflexia in both lower extremities and hypoalgesia below the T4 dermatome. Plain radiography showed normal alignment and no abnormal bony destruction was seen. Magnetic resonance imaging (MRI) of her spine showed a posterior homogeneous extradural mass of approximately 1.0 × 1.4 × 4.0cm along T4 to T7 levels with a hypointense signal on T1-weighted image (T1W), an intermediate signal on T2-weighted image (T2W) and significant enhancement in the post-contrast images (Figures 1a, 1b, 1c). On axial T2W a tumor appeared to originate from her left facet joint at T5 to T6. Abnormal marrow intensity of her left facet joint was found (Figure 2). The lesion was well circumscribed and solid. The tumor was located only in the posterior element and did not involve the vertebral body. We performed a T4 through T7 laminectomy and the tumor was totally removed. Intraoperative findings showed that the tumor had adhered to the left lamina and pedicle of T5 to T6 and had penetrated into the neural foramen of T5 and T6. The mass was confirmed to be an extradural mass in the surgical field. The gross specimen consisted of a well-capsulated, firm to hard mass measuring 1.0 × 1.5 × 4.0cm in diameter. Cut surfaces showed white-yellow tissue and a tiny bone component at the capsule (Figure 3).

**Figure 4:**
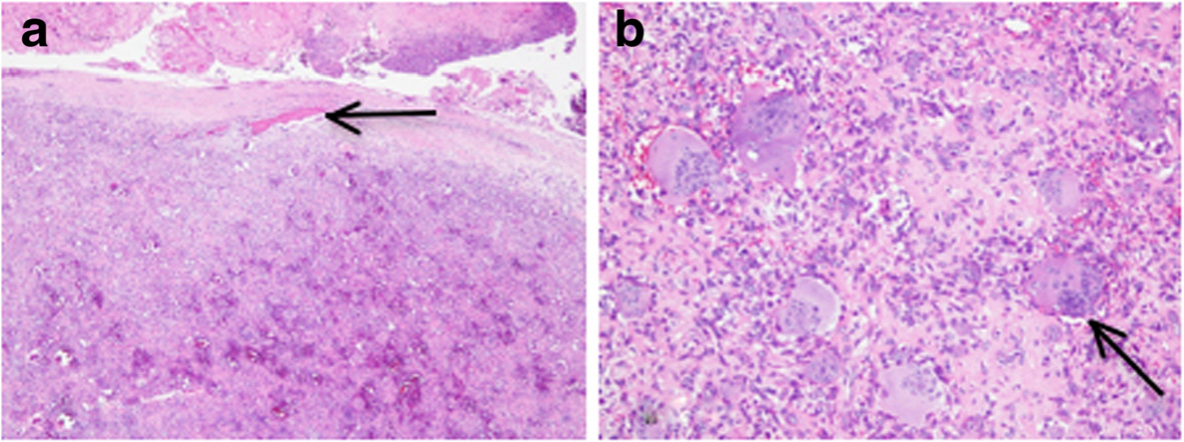
**Histopathology images of the tumor. a**. Low-power hematoxylin and eosin stain. The tumor showed a well-capsulated mass with packed polyhedral stromal cells and numerous multinucleated giant cells. There were a few tiny fragments of bone at the capsular area near the attached bone (black arrow) **b**. High-power hematoxylin and eosin stain. The main components of the tumor were polyhedral stromal cells and numerous multinucleated giant cells (black arrow). There was a lack of papillary or villiform architecture.

**Figure 5:**
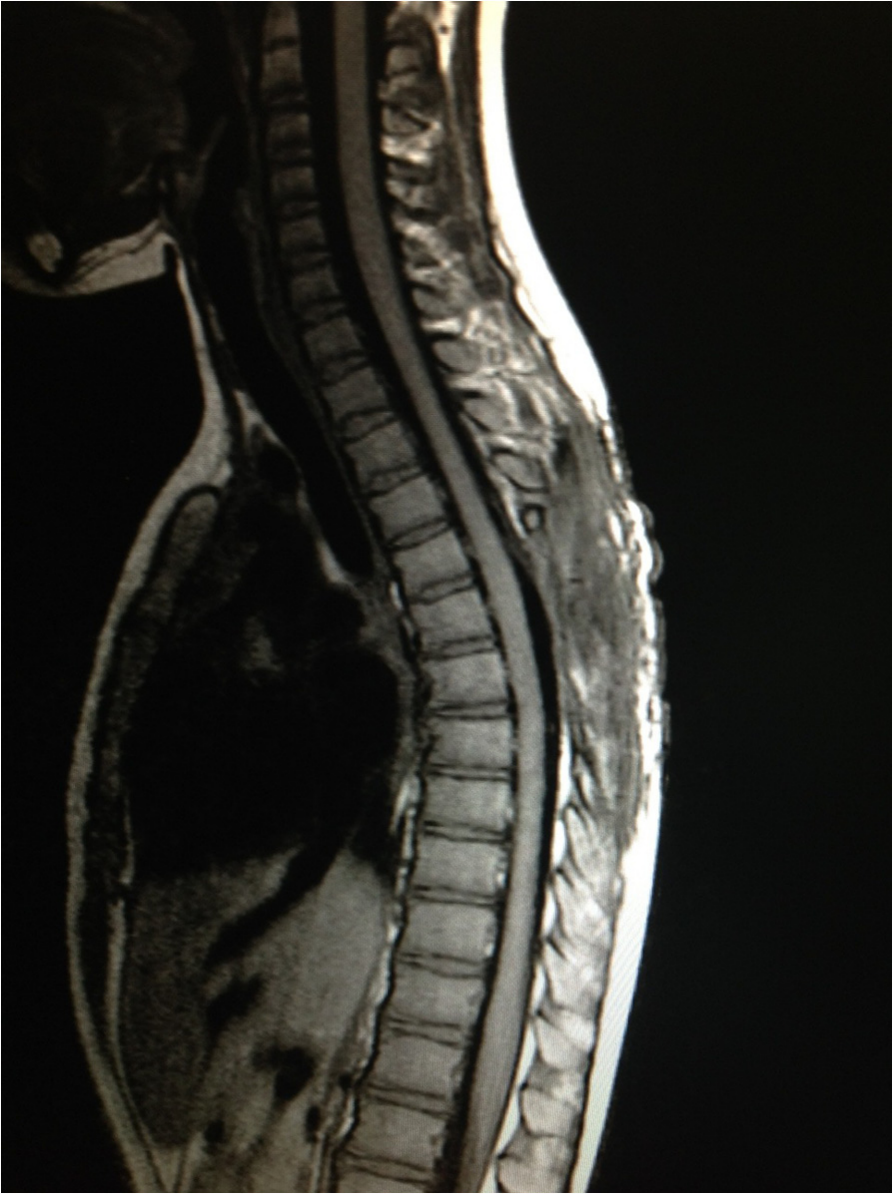
**Postoperative magnetic resonance imaging at 1 year.** Sagittal T1-weighted magnetic resonance imaging at one-year follow-up showed there was no recurrence of the tumor.

The pathological study showed that the mass was composed of packed polyhedral stromal cells and numerous multinucleated giant cells. Some areas showed hyalinized stroma. Mitotic figures were rare. The giant cells were large, and ranged from a few to 50 nuclei. There was a lack of papillary or villiform architecture. There were a few tiny fragments of bone at the capsular area near the attached bone (Figures 4a, 4b). The findings were compatible with giant cell tumor. A computed tomography (CT) chest scan showed no lung metastasis. The patient’s postoperative course was unremarkable. She did not receive adjuvant radiation therapy. The patient completely recovered from paraparesis 1 month after the operation. The MRI at 1-year follow-up showed no recurrence of the tumor (Figure 5).

## Discussion

A giant cell tumor in a facet joint of the spine is very rare. The diagnosis of giant cell tumor of the synovium of a facet joint of the spine requires a high index of suspicion. Due to the difficulty of diagnosis, the characteristic of this tumor should be studied. Radiographs of this tumor have varying appearances such as soft-tissue mass, osseous erosion and periosteal elevation. A CT scan of giant cell tumors of the tendon sheath can demonstrate bony involvement such as osseous expansion or sclerotic margin at the facet joint. An MRI commonly identifies a mass that is isointense on T1W images and has variable intensity on T2W images due to the presence of hemosiderin, cystic fluid, and hemorrhage [5], and it often shows marked enhancement after contrast administration [12]. Because these characteristics can also be found in other epidural masses such as lymphoma, meningioma, nerve sheath tumor, metastasis, and myeloma, these tumors should be considered in the differential diagnosis. Moreover, involvement of the facet joint is an important clue for the diagnosis of giant cell tumor of the synovium at the axial skeleton. But in cases that present with bony destruction at the facet joint, other primary bone lesions such as aneurysmal bone cyst, osteoblastoma and osteoclastoma should be considered in the differential diagnosis for a lesion that involves the posterior vertebral elements.

Malignant transformation of the synovial-type giant cell tumor is uncommon [14]. To date there is no standard treatment of giant cell tumor of a facet joint at the spine. The best predictor for the final outcome is the type of initial surgery performed [7]. A complete surgical resection is considered to be the best treatment [6,7]. One of the important complications is the recurrence of the tumor [15]. Early recurrence occurs when total excision is not achieved. Giannini *et al.*[6] reported a recurrence rate of 18% of giant cell tumor of the synovium at the spinal region after total tumor removal, which is comparable to PVNS of the appendicular skeleton [15]. Careful monitoring must be maintained to detect local recurrence. In cases with incomplete resection, chemotherapy with imatinib mesylate has been used recently as adjuvant treatment [4]. Some patients received radiotherapy after a total tumor removal as adjuvant treatment, but the benefits of radiation are unclear [10,15]. Because of the risks of radiation, such as neurological damage and post-radiation sarcoma, radiation is usually reserved only for inoperable cases.

## Conclusion

We reported an extremely rare case of giant cell tumor of the synovium at a thoracic facet joint in a child. Due to the difficulty of diagnosis, it is important to be aware that giant cell tumor of the synovium at a facet joint should be in the differential diagnosis of an epidural mass at the thoracic spine because its clinical and radiological features may mimic neoplastic lesions of this region. The best treatment is total excision. Due to the high recurrence rate, careful monitoring is very important.

## Consent

Written informed consent was obtained from the patient’s parents for publication of this case report and accompanying images. A copy of the written consent is available for review by the Editor-in-Chief of this journal.

## Abbreviations

CT: Computed tomography; MRI: Magnetic resonance imaging; PVNS: Pigmented villonodular synovitis; T1W: T1-weighted image; T2W: T2-weighted image.

## Competing interests

The authors declare that they have no competing interests.

## Authors’ contributions

KS was a major contributor in writing the manuscript and performed the literature review. AN performed the histological examination of the tumor. BT revised the manuscript critically. All authors read and approved the final manuscript.
